# Dopamine amplifies subthalamic long-latency responses to cortical stimulation in patients with Parkinson’s disease

**DOI:** 10.1038/s41531-026-01567-6

**Published:** 2026-10-19

**Authors:** Jean-Christin Beyer, Alessia Cavallo, Jojo Vanhoecke, Richard M. Köhler, Bassam Al-Fatly, Gerd-Helge Schneider, Katharina Faust, Gregor A. Brandt, Andrea A. Kühn, Wolf-Julian Neumann

**Affiliations:** 1 Movement Disorder and Neuromodulation Unit, Department of Neurology, Charité – Universitätsmedizin Berlin, corporate member of Freie Universität Berlin and Humboldt-Universität zu Berlin, Berlin, Germany; 2 Wyss Center for Bio and Neuroengineering - Geneva, Geneva, Switzerland; 3 Department of Neurosurgery, Charité - Universitätsmedizin Berlin, corporate member of Freie Universität Berlin and Humboldt-Universität zu Berlin, Berlin, Germany; 4 Department of Neurosurgery, Universitätsklinikum Düsseldorf, Düsseldorf, Germany; 5 Neurocure Cluster of Excellence, Charité - Universitätsmedizin Berlin, Berlin, Germany; 6 Deutsches Zentrum für Neurodegenerative Erkrankungen (DZNE), Berlin, Germany; 7 AI Hub for Care, Health and Innovation, Geneva, Switzerland

**Keywords:** Neurology, Neuroscience

## Abstract

The cortico-subthalamic hyperdirect pathway provides a rapid route for cortical influence over basal ganglia processing and is a key target of deep brain stimulation (DBS) in Parkinson’s disease (PD). However, to what degree dopamine modulates signal transmission along this pathway in humans remains unclear. Using bidirectional cortical and subthalamic electrical stimulation while invasively recording evoked responses in 15 PD patients undergoing DBS surgery, we examined cortico-subthalamic communication across dopaminergic states. Cortical stimulation evoked a short-latency subthalamic response consistent with monosynaptic hyperdirect activation that was preserved across medication conditions. In contrast, longer-latency subthalamic responses showed significant dopaminergic modulation, while cortical responses to subthalamic stimulation remained unchanged. These results reveal a temporal dissociation within the cortex–basal ganglia network, whereby fast cortical transmission is unaffected by dopamine, which in turn amplifies slower network level communication to the subthalamic nucleus. This separation of fast and delayed components provides a mechanistic framework for understanding how dopamine shapes information flow in the cortex–basal ganglia system.

## Introduction

Parkinson’s disease (PD) is the most rapidly expanding neurodegenerative condition^1,2^, characterized by the progressive degeneration of dopaminergic neurons within the substantia nigra^3^. Dopamine replacement therapy with levodopa, together with high-frequency deep brain stimulation (DBS) of the subthalamic nucleus (STN), remain the most effective interventions for alleviating PD-related motor symptoms^4,5^. Yet despite their widespread clinical use, the neural circuit mechanisms through which these treatments exert their therapeutic effects are still not fully understood^6^. One of the proposed target circuits of STN-DBS is the monosynaptic cortico-subthalamic hyperdirect pathway, which has been implicated in rapid movement cancellation, in the emergence of pathological synchronization, and as a key substrate through which STN-DBS may influence network dynamics^7–10^. Although computational models and animal experiments have highlighted the importance of this pathway^11–13^, direct causal evidence for how dopaminergic state shapes hyperdirect STN excitation in humans remains scarce^9,14^. This pathway can be probed electrophysiologically using evoked responses following electrical stimulation, which provide a time-resolved measure of signal propagation along cortico-subthalamic circuits. Our main objective is to test whether dopaminergic state differentially modulates fast versus delayed cortico-subthalamic evoked responses in humans. In this context, short-latency responses are thought to reflect fast, likely monosynaptic transmission along the hyperdirect pathway, whereas longer-latency responses are more likely to reflect polysynaptic network activity. Prior human studies have relied primarily on stimulation of the STN to measure antidromic activation with electrocorticography (ECoG), electroencephalography (EEG) or magnetoencephalography (MEG) recordings^15–19^. However, it remains unclear whether this cortical neural population activity behaves similarly to orthodromic activation and, if so, to what extent this activity can be modulated by dopamine. To address this gap, we implemented direct electrical stimulation of motor cortex delivered through externalized ECoG electrodes, during simultaneous STN local field potential (LFP) recordings in PD patients undergoing DBS implantation. Importantly, the safety and utility of ECoG-based cortical stimulation are well established in epilepsy patients, where this method is routinely used to map seizure networks, identify eloquent cortex, and characterize cortico-cortical connectivity^20,21^. Adapting this clinically validated technique to the postoperative DBS setting provides a secure and versatile platform for causal interrogation of cortical inputs to the STN, enabling repeated stimulation protocols under controlled dopaminergic conditions. We hypothesized that dopaminergic medication may differentially influence signal propagation depending on the direction of transmission. Specifically, we tested the hypothesis that dopamine selectively modulates orthodromic cortico-subthalamic transmission, while leaving STN-to-cortex responses relatively unaffected, and explicitly assessed whether dopaminergic modulation differentially affects short-latency responses, consistent with putatively monosynaptic transmission, versus long-latency responses reflecting polysynaptic network activity. Cortical responses to STN stimulation primarily reflect axonal back-propagation and cortical excitability. Orthodromic cortico-subthalamic transmission, on the other hand, depends on synaptic integration and downstream subcortical processing, both of which are strongly modulated by dopamine within the basal ganglia^22–24^. Through this multimodal, fully invasive approach, we provide the first electrophysiological evidence in humans that dopaminergic state selectively modulates orthodromic cortico-subthalamic transmission, while STN-to-cortex responses remain comparatively stable.

## Results

**Fig. 1:**
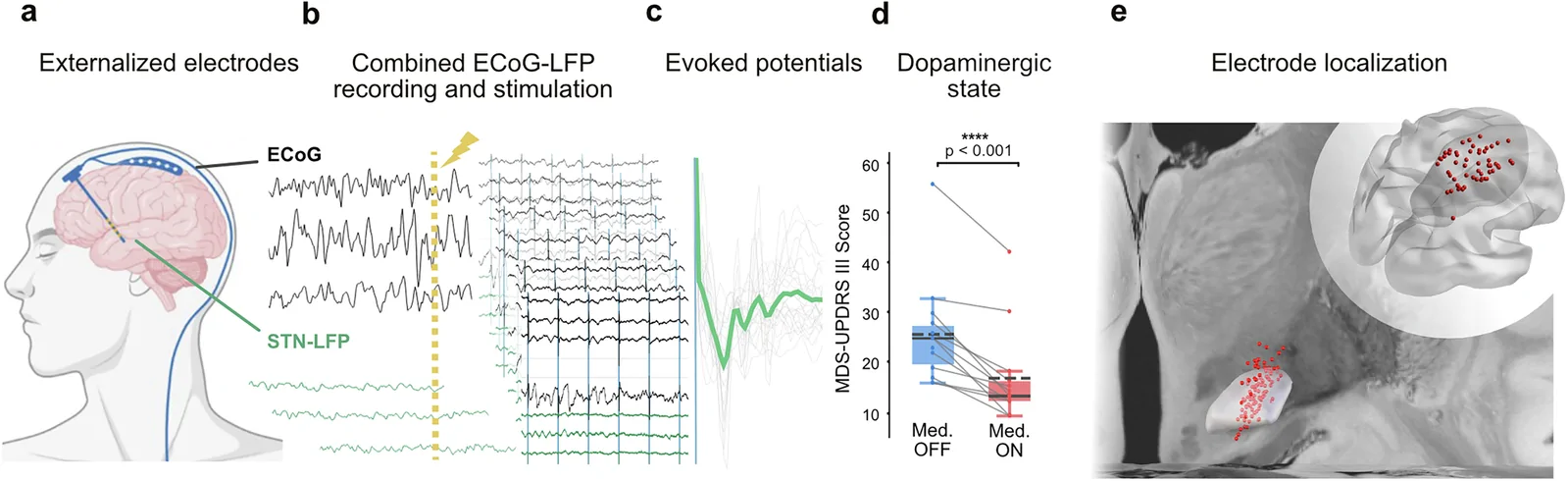
Overview of data collection and analysis. **a** ECoG strips and DBS leads were implanted in 15 patients with Parkinson’s disease. **b** Bidirectional stimulation-recording paradigms were applied through multichannel electrodes in the cortex and STN. **c** Evoked responses, time locked to stimulation, were extracted and averaged within and across patients. **d** Stimulation and recording sessions were repeated in the dopaminergic ON and OFF states to assess circuit-level dopaminergic modulation of cortico-subthalamic signal transmission. **e** Reconstructed localization of DBS and ECoG electrode contacts. DBS deep brain stimulation, ECoG electrocorticography, LFP local field potential, MDS-UPDRS Movement Disorder Society-Unified Parkinson’s Disease Rating Scale, STN subthalamic nucleus.

To causally probe dopaminergic modulation of human cortico-subthalamic signal transmission, we recruited 15 patients with PD undergoing bilateral implantation of DBS electrodes targeting the subthalamic nucleus (Supplementary Table 1). In the days following DBS lead implantation, simultaneous unilateral ECoG from sensorimotor cortex and subthalamic local field potentials (STN-LFP) were acquired through externalized leads (Supplementary Table 2). Direct electrical stimulation was delivered through ECoG electrodes positioned over the motor cortex while recording evoked responses in the STN, and vice versa. For cortical stimulation, a bipolar configuration targeting the primary motor cortex (M1) was used (contact closest to M1 and its anterior neighbour). For subthalamic stimulation, the lowermost and uppermost contacts of the DBS lead were stimulated in a bipolar configuration. This paradigm allowed quantification of both orthodromic cortico-subthalamic drive following cortical stimulation and antidromic cortical response following STN stimulation in the same individuals. Stimulation was performed in both dopaminergic ON and OFF states, with OFF-state recordings obtained after withdrawal of dopaminergic medication for at least 12 hours with a clinically meaningful improvement after intake (MDS-UPDRS III OFF: 25.9 ± 9.7; ON: 16.6 ± 8.9; *p* < 0.001) (Fig. 1), enabling within-patient assessment of dopamine-dependent changes in pathway excitability. For cortical stimulation, paired analyses were performed in patients with both dopaminergic ON and OFF recordings (*n* = 10), while 2 patients contributed only ON and 3 patients only OFF data. For STN stimulation, 3 patients were recorded only in the medication ON condition, while 3 patients only participated in the medication OFF condition, yielding 9 paired datasets.

After localization of motor-cortical ECoG- and STN-DBS electrodes and signal quality confirmation, all multisite recordings were processed to extract evoked responses, representing neural activity elicited by and time-locked to individual stimulation pulses. For each stimulation direction, we identified short-, medium-, and long-latency components and quantified their amplitudes, as well as temporal and spatial structure.

### Cortical stimulation evokes short-latency subthalamic responses consistent with hyperdirect pathway activation

**Fig. 2:**
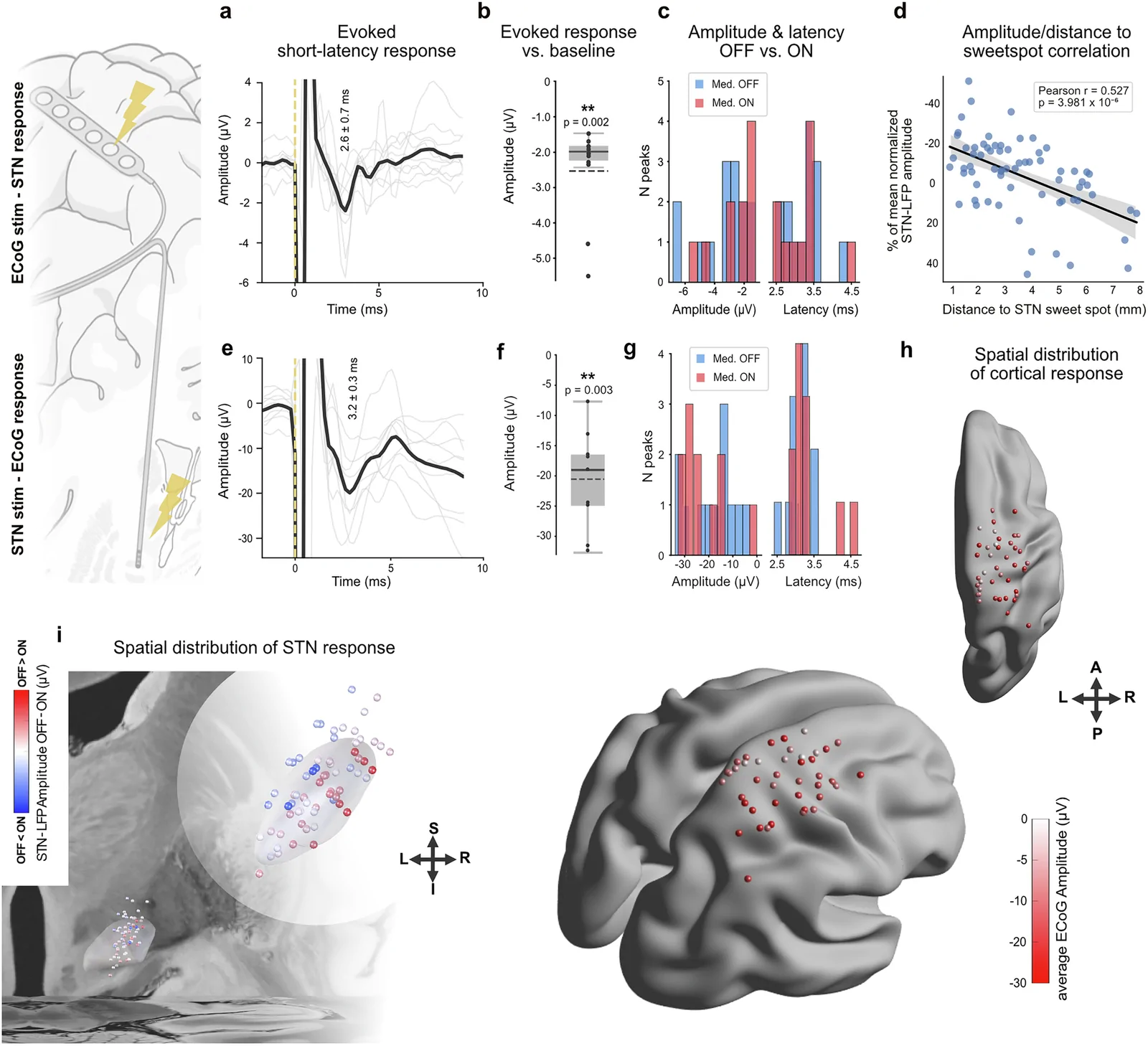
Cortical and subthalamic stimulation evoke concordant short-latency responses. **a** Mean (thick dark grey line) and single-patient (light grey lines) short-latency STN responses following ECoG stimulation, averaged across ON and OFF medication conditions (*n* = 10) with the first deflection detectable at 2.6 ± 0.7 ms. **b** Amplitude of the first deflection significantly differs from baseline activity (*p* = 0.002). **c** Comparison of STN-LFP amplitude and latency between dopaminergic OFF and ON medication states shows no significant dopaminergic modulation of the short-latency STN response (mean amplitude OFF: −3.3 ± 1.7 µV; ON: −2.6 ± 1.4 µV, paired comparison, *n* = 10, *p* = 0.06; mean latency OFF: 2.7 ± 0.6 ms; ON: 2.6 ± 0.8 ms, paired comparison, *n* = 10, *p* = 0.36). **d** STN contacts closer to the STN sweet spot exhibited more negative evoked STN-LFP amplitudes, with amplitudes becoming progressively less negative with increasing distance (Pearson r = 0.527, *p* = 3.981 × 10⁻^6^). The y-axis is inverted for visualization. **e** Mean (thick dark grey line) and single-patient (light grey lines) short-latency ECoG responses following STN stimulation, averaged across ON and OFF medication conditions (*n* = 9), with the first deflection detectable at 3.2 ± 0.3 ms. **f** Amplitude of the first ECoG deflection is significantly different from baseline activity (*p* = 0.003). **g** Comparison of ECoG amplitude and latency between dopaminergic OFF and ON medication states shows no significant difference in the short-latency time window (mean amplitude OFF: −18.2 ± 9.7 μV, ON: −22.1 ± 9.8 μV, paired comparison, *n* = 9, *p* = 0.367; mean latency OFF: 3.1 ± 0.3 ms, ON: 3.3 ± 0.6 ms, paired comparison, *n* = 9, *p* = 0.288). **h** ECoG channels showing the highest average ECoG Amplitude are mainly located in the sensorimotor cortex. **i** DBS channels exhibiting the largest differences in STN responses between OFF and ON medication conditions, with higher amplitudes in the OFF state, are mainly located in the dorsolateral STN. A anterior, ECoG electrocorticography, I inferior, L left, LFP local field potential, P posterior, R right, S superior, STN subthalamic nucleus, OFF and ON refers to OFF dopaminergic medication/ON dopaminergic medication.

**Fig. 3:**
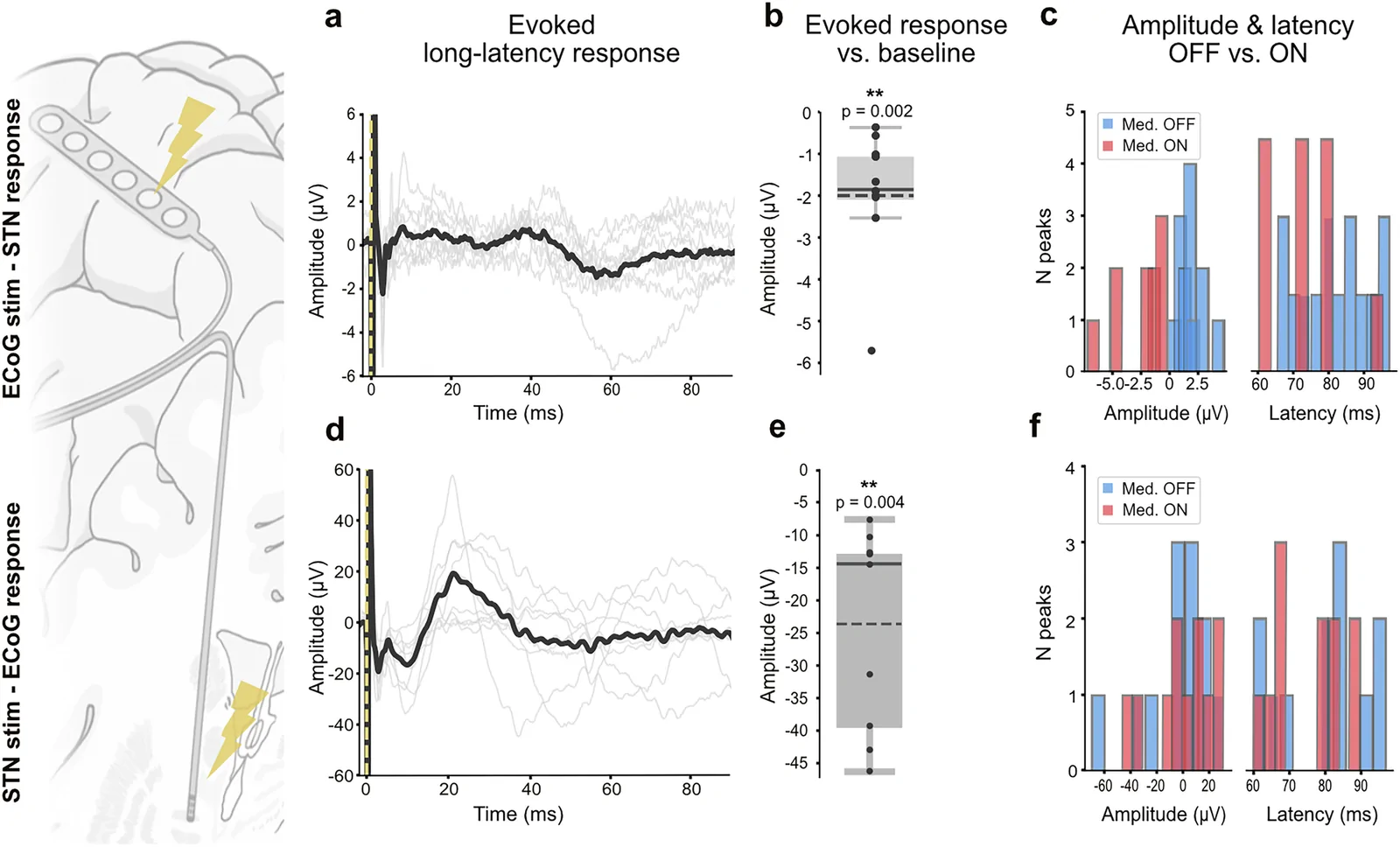
Cortical and subthalamic long-latency responses. **a** Mean (thick dark grey line) and single-patient (light grey lines) STN response following ECoG stimulation averaged across ON- and OFF medication conditions (*n* = 10). **b** Amplitude of the STN long-latency response significantly differs from baseline activity (*p* = 0.002). **c** STN-LFP amplitudes in the OFF medication condition (mean 1.5 ± 1.2 µV) are characterized by positive deflections, whereas the ON medication condition shows significantly more negative deflections (mean −2.2 ± 2.2 µV, paired comparison, *n* = 10, *p*  0.002), while latency did not significantly differ between conditions (OFF: 81.5 ± 10.2 ms; ON: 73.6 ± 10.7 ms, paired comparison, *n* = 10, *p* = 0.09). **d** Mean (thick dark grey line) and single patient (light grey lines) ECoG response following STN stimulation averaged across ON- and OFF medication conditions (*n* = 9). **e** Amplitude of the ECoG long-latency response is significantly different from baseline activity (*p* = 0.004). **f** ECoG amplitude and latency are not significantly modulated by dopaminergic medication (mean amplitude OFF: −3.9 ± 26.8 μV, ON: −0.2 ± 24.2 μV, paired comparison, *n* = 9, *p* = 0.059; mean latency OFF: 78.7 ± 10 ms, ON: 75.5 ± 8 ms, paired comparison, *n* = 9, *p* = 0.258). ECoG electrocorticography, LFP local field potential, STN subthalamic nucleus, OFF and ON refers to OFF dopaminergic medication/ON dopaminergic medication.

Direct electrical stimulation of the motor cortex reliably elicited a sequence of time-locked responses in the STN, beginning with a prominent short-latency component consistent with activation of the cortico-subthalamic hyperdirect pathway^25^. Across patients, cortical stimulation produced an earliest STN response at 2.6 ± 0.7 ms, followed by medium-latency components at 10.1 ± 1.9 ms, 23.9 ± 3.4 ms and 38.9 ± 3.5 ms (Figs. 2a and 3a). The earliest deflection significantly differed from baseline activity across all patients (*p* = 0.002) (Fig. 2b). With a consistent morphology across subjects and being evident in nearly all recorded STN channels, the short-latency response demonstrates robust and reproducible engagement of fast cortico-subthalamic projections, indicative of reliable orthodromic monosynaptic hyperdirect pathway activation. Next, we compared STN responses evoked by cortical stimulation with cortical responses elicited by STN stimulation in order to assess whether response latencies were consistent across stimulation directions. STN stimulation reliably elicited cortical evoked responses across patients, with identifiable short-latency components emerging at 3.2 ± 0.3 ms followed by medium-latency deflections at 6.4 ± 1.1 ms, 11.5 ± 1.8 ms and 23.2 ± 2.4 ms (Figs. 2e and 3d). Consequently, the cortical short-latency component did not significantly differ from the earliest STN response following cortical stimulation (Wilcoxon signed-rank test, *p* = 0.109). Short-latency cortical deflections evoked by STN stimulation were significantly different from baseline (*p* = 0.003), demonstrating that cortical response reliably reflects stimulus-locked physiological activity (Fig. 2f). Cortical deflections were spatially consistent across the sensorimotor ECoG contacts and exhibited stable morphology across stimulation intensities and runs. Across subjects, the amplitude and latency of STN responses evoked by cortical stimulation did not correlate with those of cortical responses evoked by STN stimulation, indicating that antidromic and orthodromic responses underlie different neurophysiological dynamics.

In both stimulation directions, the amplitude and morphology of the first peak remained stable when stimulation was performed in the dopaminergic OFF condition and after levodopa administration. The amplitude of the first negative STN peak occurring at ~2–4 ms was not significantly different between conditions (mean amplitude OFF: −3.3 ± 1.7 µV; ON: −2.6 ± 1.4 µV, paired comparison, *n* = 10, *p* = 0.06), showing only a weak difference with a less negative deflection in the dopaminergic ON state. STN peak latencies were nearly identical in ON and OFF medication states (OFF: 2.7 ± 0.6 ms; ON: 2.6 ± 0.8 ms, paired comparison, *n* = 10, *p* = 0.36).

Across patients, contacts exhibiting the largest differences in short-latency STN-LFP amplitudes between OFF and ON medication states were mainly located within the dorsolateral region of the STN, whereas more ventromedial contacts showed smaller differences (Fig. 2i). Moreover, evoked STN-LFP responses were largest near the STN sweet spot and progressively became less negative with increasing distance (Pearson r = 0.527*, p* = 3.981 *×* 10⁻^6^) (Fig. 2d). Distances were computed relative to a dorsolateral STN region based on previously published probabilistic mapping of clinically effective stimulation sites in MNI space^26^. Consistent with this spatial pattern, the contact with the largest short-latency STN-LFP amplitude frequently corresponded to the clinically selected stimulation site for chronic DBS therapy, whereas long-latency response amplitudes did not show a clinical correlation. To quantify this relationship, the data-driven electrode selection (maximum evoked amplitude of the first deflection) was compared with the stimulation level used for chronic stimulation, determined using standard clinical programming at either the 12-month follow-up (MFU) visit (*n* = 7) or, when this visit had not yet occurred, the 3-MFU visit (*n* = 8). Agreement was defined at the contact level. A “match” was defined as the contact with the largest evoked response being located on the same contact level as the clinically selected stimulation contact(s). Using this definition, the maximal-evoked contact was located on the clinically selected level in 12 out of 15 patients (80%). Under the null hypothesis of equal probability of selecting either of the four contact levels (*p* = 0.25), this exceeded chance level (two-sided binomial test, *p* < 0.001). At the level of individual segments, agreement was more variable, reflecting the frequent use of multi-segment stimulation within a given level.

### Subthalamic but not cortical long-latency responses are modulated by dopamine

In addition to the short-latency cortico-subthalamic responses, we next examined long-latency responses to subthalamic and cortical stimulation in more detail. Cortical stimulation revealed robust orthodromic long-latency responses in the STN-LFP signals, significantly deviating from baseline activity (*p* = 0.002) between 40 and 100 ms, with a mean peak latency of 79.9 ± 8.9 ms (Fig. 3a). Similarly, STN stimulation reliably elicited cortical responses across patients, emerging at an average of 77.3 ± 7.1 ms (Fig. 3d) with significant differentiation from baseline (*p* = 0.004).

**Fig. 4:**
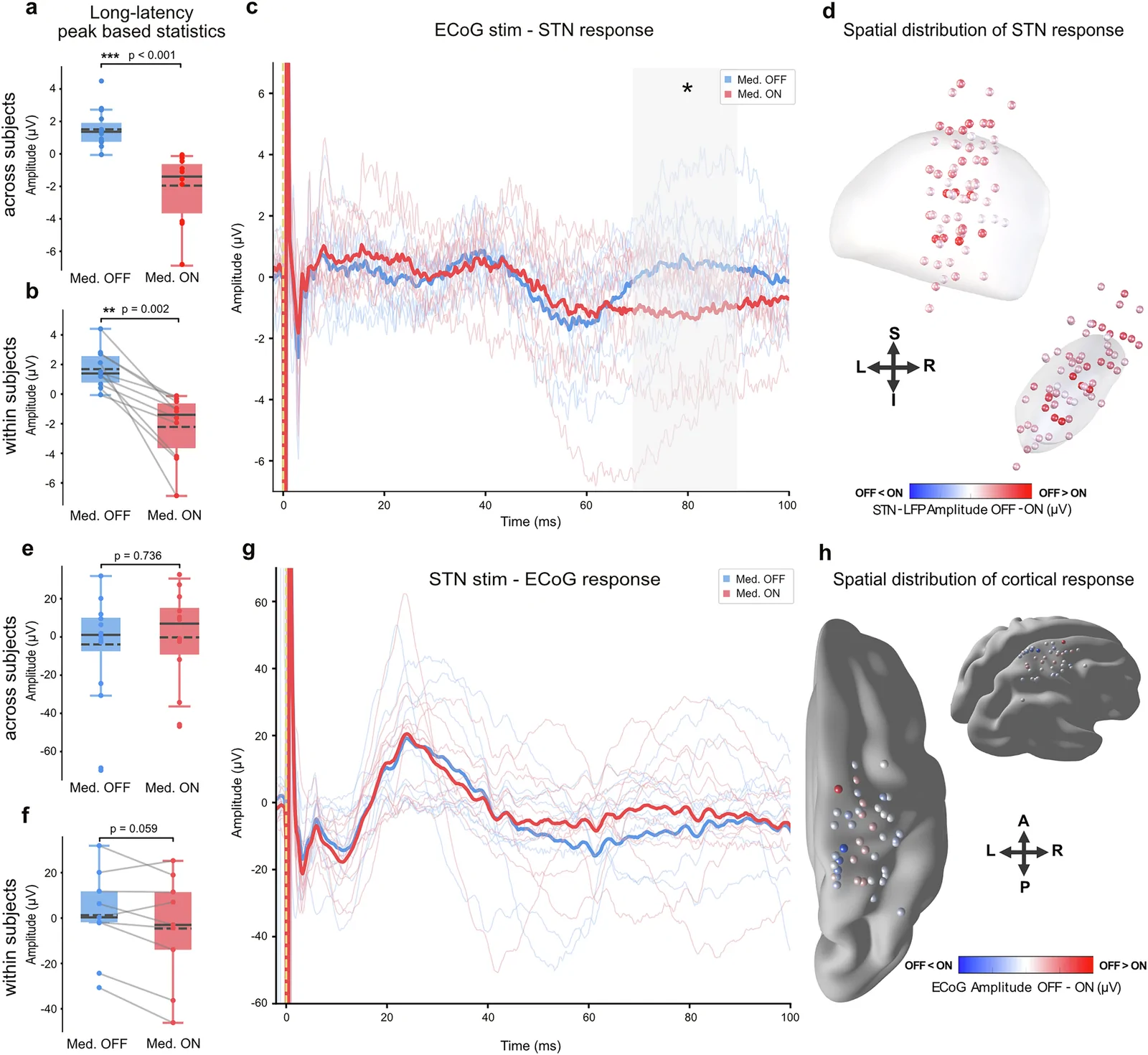
Dopaminergic modulation of subthalamic and cortical long latency responses. **a**–**c** Temporally resolved cluster-based permutation testing reveals a shorter-lasting response in the dopaminergic OFF state compared to a more prolonged negative deflection in the ON state during the time window of 65–85 ms (OFF: 1.5 ± 1.2 µV, ON: −2.2 ± 2.2 µV, unpaired comparison: *n* = 13 OFF vs *n* = 12 ON, *p* < 0.001; paired comparison: *n* = 10, *p* = 0.002). **d** The largest amplitude differences, with faster return to baseline in the OFF dopaminergic condition and more sustained negative deflections in the ON condition, are observed in DBS channels located in the dorsolateral region of the STN. **e**–**g** Cortical response to STN stimulation shows no significant dopaminergic modulation of amplitude using temporally resolved cluster-based permutation testing (OFF: −3.9 ± 26.8 μV, ON: −0.2 ± 24.2 μV, *p* = 0.736 for unpaired comparison *n* = 12 OFF vs *n* = 12 ON, *p* = 0.059 for paired comparison, *n* = 9). **h** ECoG channels showing the largest OFF-ON amplitude differences, with higher amplitudes in the OFF state are located in the motor cortex. A anterior, ECoG electrocorticography, I inferior, L left, LFP local field potential, P posterior, R right, S superior, STN subthalamic nucleus, OFF and ON refers to OFF dopaminergic medication/ON dopaminergic medication. Shaded grey area indicates cluster of significant amplitude difference between ON/OFF conditions; *p  <  0.05.

In contrast to the short-latency peak, the long-latency subthalamic response had a significantly more negative amplitude in the medication ON state, which we refer to as amplification, defined as an increase in both the duration and negativity of the response compared to the OFF state. In the OFF state, the late STN-LFP peak reached 1.5 ± 1.2 µV, compared to −2.2 ± 2.2 µV in the ON state (unpaired comparison: *n* = 13 OFF vs *n* = 12 ON, *p* < 0.001; paired comparison: *n* = 10, *p* = 0.002; Fig. 4a, b). This difference was confirmed using a cluster-based permutation test (corrected for multiple comparisons, 1000 iterations) with a longer lasting negative evoked response in the ON state, which was temporally restricted to the long latency time window and apparent between 65–85 ms (Fig. 4c). To control for differences in stimulation frequency (1 Hz (*n* = 4) vs 5 Hz (*n* = 11)), the analysis was repeated including only subjects stimulated at 5 Hz. The results remained significant, confirming that the main findings were not driven by stimulation frequency (Supplementary Fig. 1). To further investigate the potential dopaminergic effect on the temporal dynamics of signal transmission, we analysed whether the temporal delay, measured as peak latency, varied between OFF and ON states. The latency of the late peak did not differ significantly between conditions (OFF: 81.5 ± 10.2 ms; ON: 73.6 ± 10.7 ms, paired comparison, *n* = 10, *p* = 0.09), indicating that dopamine depletion modulates response amplitude rather than the temporal structure of the underlying circuit dynamics.

Regarding the spatial distribution of the long-latency response, deflections with larger amplitudes in the OFF compared to the ON medication condition were again observed at contacts located within the dorsolateral STN (Fig. 4d). Having established that dopamine selectively modulates long-latency subthalamic responses to cortical stimulation, we next investigated whether a similar dopaminergic influence is present in cortical responses following STN stimulation. While short-latency cortical responses to STN stimulation are consistent with antidromic activation, responses at longer latencies (~ 77 ms) are unlikely to reflect direct antidromic conduction and instead may arise from polysynaptic network activity. Comparing orthodromic cortico-subthalamic responses with cortical responses following STN stimulation therefore allows us to assess whether dopaminergic modulation generalizes across stimulation directions, despite differences in the underlying response mechanisms.

In contrast to the descending cortico-STN pathway, none of the cortical components evoked by STN stimulation showed significant modulation by dopaminergic state. Short-latency peaks displayed comparable amplitudes and latencies in the ON and OFF medication conditions (ON: −22.1 ± 9.8 μV, OFF: −18.2 ± 9.7 μV, *p* = 0.367; ON: 3.3 ± 0.6 ms, OFF: 3.1 ± 0.3 ms, *p* = 0.288, paired comparison, *n* = 9), indicating preserved early subthalamocortical conduction regardless of dopamine availability. Likewise, long-latency cortical deflections did not significantly differ between medication states (ON: −0.2 ± 24.2 μV, OFF: −3.9 ± 26.8 μV, *p* = 0.059; ON: 75.5 ± 8 ms, OFF: 78.7 ± 10 ms, *p* = 0.258, paired comparison, *n* = 9) (Fig. 4e–g).

This absence of dopaminergic influence persisted both at the single-channel level and when averaging across sensorimotor ECoG sites, as well as in across subjects and within subjects comparison (*p* = 0.736 for unpaired comparison *n* = 12 OFF vs *n* = 12 ON, *p* = 0.059 for paired comparison, *n* = 9), and it remained stable when quantifying responses using temporally resolved cluster-based permutation statistics.

### Motor responses during cortical stimulation occur in a subset of patients

**Fig. 5:**
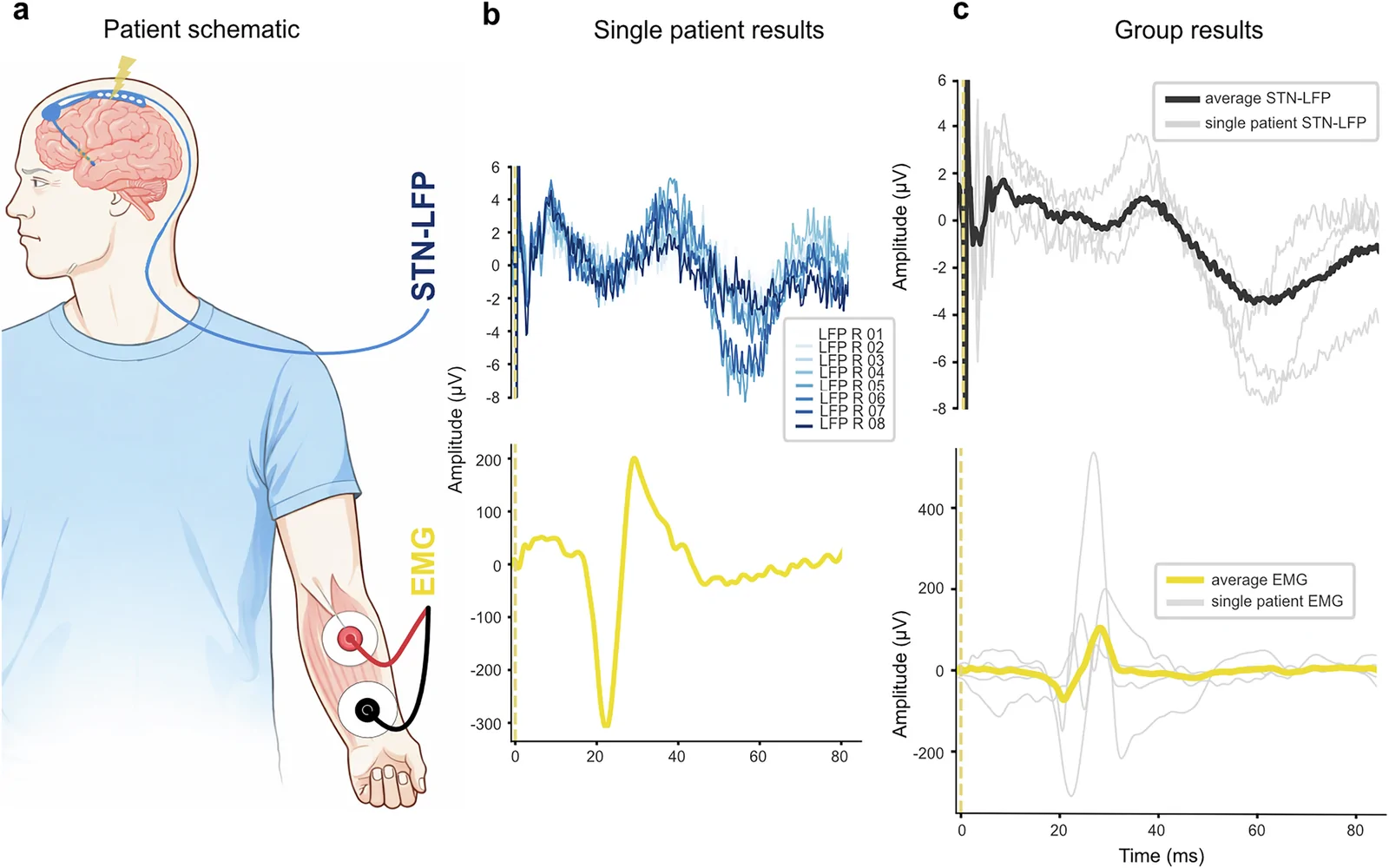
Motor response evoked by cortical stimulation. **a** Patient schematic. **b** Single-patient example of STN-LFP and EMG responses. **c** Upper panel: average (thick dark grey line) and single-patient (light grey lines) STN-LFP responses of patients showing a motor response following direct cortical stimulation (*n* = 5, including 4 paired datasets), averaged across ON and OFF medication conditions (peak short-latency STN-LFP amplitude with EMG response was −3.6 ± 1.8 μV (ON) vs −4.5 ± 2 μV (OFF) (paired comparison, *n* = 4, *p* = 0.50)). Peak long-latency STN-LFP amplitude was −4 ± 3 μV (ON) vs 1.5 ± 0.5 μV (OFF) (paired comparison, *n* = 4, *p* = 0.25). Lower panel: average (thick yellow line) and single-patient (light grey lines) EMG responses averaged across ON and OFF medication conditions, occurring at a latency of 24.1 ± 7.1 ms (ON) vs 28.9 ± 16.4 ms (OFF) (paired comparison, *n* = 4, *p* = 0.75). Peak-to-peak EMG amplitudes were 571.7 ± 415.2 μV (ON) vs 126.8 ± 79.5 μV (OFF) (paired comparison, *n* = 4, *p* = 0.25), showing substantial inter-subject variability. EMG electromyography, LFP local field potential, STN subthalamic nucleus; OFF and ON refer to OFF and ON dopaminergic medication states.

In a subset of five patients, cortical stimulation elicited measurable peripheral motor activation, detectable both via surface EMG recordings and visible muscle contractions. These motor responses occurred at latencies consistent with corticospinal conduction^27^, emerging at 24.1 ± 7.1 ms (ON) vs 28.9 ± 16.4 ms (OFF), paired comparison, *n* = 4, *p* = 0.75 after stimulation. These responses primarily involved contralateral distal upper limb muscles, most frequently manifesting as wrist extension. Peak-to-peak EMG amplitudes were 571.7 ± 415.2 μV (ON) vs 126.8 ± 79.5 μV (OFF), paired comparison, *n* = 4, *p* = 0.25, reflecting substantial variability across subjects (Fig. 5). The presence of EMG activity was tightly coupled to stimulation intensity and electrode position, suggesting direct engagement of descending motor pathways in patients with closer proximity between the ECoG electrode and primary motor cortex.

Importantly, the emergence of peripheral motor responses did not correspond to systematic changes in subthalamic evoked potentials. Short- and long-latency STN components in patients with EMG activity were comparable in amplitude, morphology, and medication-related modulation to those observed in patients without motor responses (peak short-latency STN-LFP amplitude with EMG response: −3.6 ± 1.8 μV (ON) vs −4.5 ± 2 μV (OFF), paired comparison, *n* = 4, *p* = 0.50; without EMG response: −2.2 ± 1.1 μV (ON) vs −2.6 ± 1 μV (OFF), paired comparison, *n* = 6, *p* = 0.22; ON condition short-latency STN-LFP amplitude EMG response group (*n* = 4) vs no EMG response group (*n* = 8): *p* = 0.11 (unpaired comparison), OFF condition short-latency STN-LFP amplitude EMG response group (*n* = 5) vs no EMG response group (*n* = 8): *p* = 0.08 (unpaired comparison)).

Similarly, long-latency STN responses during cortical stimulation remained unaffected by whether cortical stimulation in the same patient elicited peripheral motor activity (peak long-latency STN-LFP amplitude with EMG response: −4 ± 3 μV (ON) vs 1.5 ± 0.5 μV (OFF); without EMG response: −1.4 ± 1.5 μV (ON) vs 1.8 ± 1.6 μV (OFF); ON condition long-latency STN-LFP amplitude EMG response group (*n* = 4) vs no EMG response group (*n* = 8): *p* = 0.27 (unpaired comparison), OFF condition long-latency STN-LFP amplitude EMG response group (*n* = 5) vs no EMG response group (*n* = 8): *p* = 1 (unpaired comparison)), suggesting that the observed evoked subthalamic potentials reflect cortico-subthalamic communication rather than back-propagation of motor output and sensory feedback. Amplitude and latency of short- and long-latency STN responses did not correlate with EMG amplitude or latency (all *p* > 0.05).

## Discussion

In our study, we establish a bidirectional stimulation-recording paradigm that enables direct physiological quantification of cortico-subthalamic communication in patients with PD. This work builds on a substantial body of animal and tractography research. In particular, cortical stimulation studies in non-human primates have demonstrated that cortical influence on the STN is conveyed through a fast, monosynaptic hyperdirect projection as well as slower, polysynaptic routes involving the striatum and indirect pathway^28–31^.

Against this background, our results provide direct electrophysiological evidence of dual cortico-subthalamic signalling in humans and extend prior work by resolving its temporal and dopaminergic specificity within the same individuals. By stimulating the motor cortex and the STN while recording bidirectional evoked responses across medication states, we identify two separable components, including a short-latency response that is preserved across dopaminergic states and a later component that demonstrates greater sensitivity to dopaminergic modulation. While these findings are consistent with a functional distinction between fast monosynaptic hyperdirect transmission and slower network-level circuit activity, the precise mechanisms underlying the later component cannot be determined directly from the present data.

The earliest responses to cortical stimulation emerged at ~2–4 ms in the STN and were mirrored by comparably rapid antidromic cortical potentials following STN stimulation. The tight temporal correspondence and consistent morphology of these reciprocal responses across channels and subjects provide convergent electrophysiological evidence consistent with direct monosynaptic cortico-subthalamic connectivity in humans, complementing prior reports of short-latency cortical potentials evoked by STN stimulation^7,25,29^. Notably, the invariance of this short-latency component across ON and OFF dopaminergic states supports its interpretation as a hard-wired monosynaptic transmission. Given the short latency of these responses, a contribution of stimulation artefact or volume conduction cannot be entirely excluded. However, the responses consistently occurred after the end of the stimulation artefact, remained unchanged following polarity reversal of the bipolar stimulation, and showed spatial specificity, supporting a physiological origin of the recorded signals. Recent work has highlighted the utility of phase-based approaches such as Inter-trial Phase Coherence (ITPC), which quantifies the consistency of phase alignment across repeated stimulations, and the directed Phase Lag Index (dPLI), which characterizes the predominant direction of phase relationships while reducing the influence of zero-lag volume conduction^32^. Such approaches could provide valuable complementary information regarding the consistency and directionality of cortico-subthalamic communication. In the present study, however, signal propagation was probed using direct cortical and subthalamic stimulation paradigms that experimentally imposed the direction of activation and allowed time-resolved characterization of the resulting evoked responses. Future studies combining stimulation-evoked analyses with phase-based measures may therefore provide a more comprehensive characterization of cortico-subthalamic information flow.

In contrast, responses at longer latencies were markedly modulated by dopaminergic state, consistent with engagement of polysynaptic or network-level processing within the basal ganglia. Prior work in non-human primates has shown that delayed STN responses to cortical stimulation depend on striatal recruitment and indirect pathway signalling^28^, supporting the interpretation that this later component may reflect dopamine-sensitive circuit dynamics. However, given the indirect nature of the present measurements, these findings should be interpreted cautiously, as the specific pathways and synaptic mechanisms contributing to the long-latency response cannot be directly resolved. Together, these findings suggest a functional separation between preserved fast cortico-subthalamic transmission and a slower, dopamine-dependent network response, clarifying which elements of cortico-basal ganglia communication remain preserved and which become dysregulated in the Parkinsonian state.

The dopaminergic amplification of long-latency STN responses in our human data may reflect altered network gain within basal ganglia circuits, potentially influencing susceptibility to recurrent excitation or feedback loops, while leaving fast cortico-subthalamic transmission largely intact. The shorter-lasting subthalamic response to cortical stimulation with faster return to baseline in the OFF state could in contrast enable exaggerated beta-band resonance commonly observed in PD^33^, as pathological synchrony often relies on recurrent network dynamics. While this framework provides a plausible interpretation of the observed effects, it remains inferential and requires further investigation. More broadly, these findings may help reconcile diverse observations on cortico-basal ganglia communication in PD, suggesting that therapeutic interventions differentially engage distinct temporal pathways. This interpretation is consistent with recent work that differentiates mesoscale network effects of dopamine versus DBS, which propose that dopamine and DBS can produce overlapping but pathway-specific modulatory effects on network synchrony and population dynamics, implying that therapeutic stimulation and dopaminergic state act on overlapping circuitry but with different mesoscale signatures^34^. More importantly, the dissociation of pathway effects was also proposed on a behavioural level, where dopamine did not affect hyperdirect processing, while DBS did^35^. Therapeutically, this positions the STN, and specifically its indirect pathway afferents, as a critical modulator of pathological basal ganglia dynamics in PD. Interventions that modulate long-latency, network-level gain, for example via pharmacological strategies or DBS, could normalize aberrant STN responsiveness while preserving physiologically necessary fast cortical drive.

Dopamine-dependent modulation was largely restricted to descending cortico-subthalamic signalling, whereas cortical responses to STN stimulation remained stable across medication states. This directional asymmetry likely reflects differences in dopaminergic innervation between cortex and basal ganglia. Cortical pyramidal neurons receive sparse dopaminergic input. On the cortex level dopamine primarily modulates excitability through D1-like receptors, resulting in subtle, state-dependent effects in cortical responsiveness. In contrast, the basal ganglia are densely innervated by midbrain dopaminergic projections. Here, dopamine exerts strong bidirectional effects through D1 and D2 receptors. Loss of dopamine therefore increases responsiveness of STN and indirect-pathway structures, enhances excitatory drive through the STN-GPe loop, and amplifies the gain of cortically evoked responses^22,23,36^. Consistent with these mechanisms, the dopaminergic modulation of long-latency responses observed in our data suggests that the recorded signals are embedded within a biologically responsive system. However, this observation should not be interpreted as definitive evidence against all non-neuronal contributions, as the electrical properties of extracellular tissue are themselves dynamically shaped by neuronal activity, neurotransmitter concentrations, and glial processes and may influence extracellular signal propagation and filtering^37^.

In a subset of patients, cortical stimulation elicited peripheral motor activation detectable by EMG. The presence or absence of these motor responses did not influence the amplitude, morphology, or dopaminergic modulation of STN-evoked potentials, indicating that the central responses were not confounded by peripheral back-propagation from descending motor output. The EMG latencies observed here (∼24–30 ms) are consistent with established cortico-spinal conduction times reported in human motor-evoked potential studies using transcranial magnetic stimulation (TMS)^27,38^. The dissociation between peripheral motor activation and STN responses confirms that cortico-subthalamic evoked potentials arise within central circuits upstream of the corticospinal tract. Even at stimulation intensities reaching the motor threshold, STN responses remained stable, reinforcing the validity of ECoG-based stimulation for probing cortico-subthalamic communication^18,25^. However, these findings should be interpreted with caution given the relatively small number of patients exhibiting EMG responses. Importantly, this study aimed to understand the relationship between the presence of movement and subthalamic activation, rather than establishing precise anatomical specificity between cortical sites and EMG activity. While additional connectivity analyses could further elucidate these motor circuits, the transient nature of the evoked EMG responses and the limited number of patients exhibiting these responses in our data pose significant analytical limitations for such methods. These findings should therefore be interpreted with caution, and we propose directed connectivity as an important avenue for future studies with larger cohorts.

The spatial distribution of evoked responses provides further insight into the circuit mechanisms underlying dopamine-sensitive STN activity. For both early and late components, contacts exhibiting the strongest negative amplitudes were consistently localized to the dorsolateral STN, the canonical motor territory^30,39^. This region-specific predominance aligns with the area’s dense motor cortical inputs and its susceptibility to pathological synchronization in PD^40^. This convergence of anatomical and physiological findings suggests that dopamine selectively increases the gain of indirect pathway input within the dorsolateral STN and reinforces this region as the primary therapeutic target for both DBS and dopaminergic circuit modulation.

In this regard, our findings are consistent with prior work showing that the amplitudes of short-latency cortical responses to STN stimulation are strongly aligned with clinically effective DBS contacts^25^. This has direct clinical implications, as the contact location that produced the largest short-latency STN-LFP amplitude matched the clinically chosen therapeutic DBS contact level in 80% of cases. The probability of this alignment occurring by chance was exceedingly small (*p* < 0.001), underscoring that these early evoked responses may capture aspects of pathway engagement, such as proximity to hyperdirect fibre terminations or to nodes of increased cortico-basal ganglia coupling that are preferentially modulated by stimulation. This highlights their potential utility as intraoperative or postoperative biomarkers to guide contact selection and reduce reliance on prolonged clinical testing. However, this translational interpretation should be considered with caution, as it is based on a simplified choice model and limited sample size, and requires further validation in larger, prospective studies.

By combining bidirectional stimulation and multisite recordings within the same individuals, our results demonstrate that human hyperdirect pathway signalling can be dissected with millisecond precision. They reveal a temporally and mechanistically dissociable architecture comprising a fast, likely monosynaptic hyperdirect volley that is dopamine insensitive and a slower, polysynaptic network response that shows greater susceptibility towards dopaminergic modulation. While the precise circuit mechanisms underlying the latter remain to be fully established, this dissociation provides insight into the circuit-level alterations associated with Parkinsonian motor dysfunction and identifies long-latency cortico-subthalamic responses as candidate markers of pathological network state with potential relevance for future neuromodulation strategies. By integrating cortical and subcortical stimulation within the same individuals, this work lays the foundation for future mechanistic studies of directional information flow in movement disorders.

## Methods

### Participants

Fifteen patients (age 59.4 ± 10.5, 13 male) diagnosed with idiopathic PD undergoing bilateral implantation of DBS electrodes targeting the STN were enrolled at the Department of Neurosurgery, Charité – Universitätsmedizin Berlin (Supplementary Table 1). All patients exhibited a predominantly akinetic-rigid motor phenotype and met established clinical criteria for DBS therapy. For research purposes, a subdural ECoG electrode strip (Ad-Tech) was implanted unilaterally over the sensorimotor cortex. Due to the limited cohort size, sex and gender were not considered as covariates in the study design.

For cortical stimulation, 10 patients had paired ON/OFF medication recordings, while 2 contributed ON-only and 3 OFF-only data. For STN stimulation, 3 patients were recorded only in the medication ON condition, while 3 only participated in the medication OFF condition, resulting in 9 paired datasets (Supplementary Table 2). The order of ON and OFF medication recordings was not counterbalanced, as recordings were performed in the early postoperative period, and prioritizing the ON-medication state first minimized patient burden and discomfort during recovery.

### Ethics declaration

All procedures were approved by the Ethics Committee of Charité – Universitätsmedizin Berlin (approval number: EA2/129/17, EA1/128/23) and conducted in accordance with the Declaration of Helsinki. All patients provided written informed consent to participate in the research and to the implantation of the temporary ECoG electrode strip. Data collection, storage, and processing conformed to the General Data Protection Regulation of the European Union.

### DBS and ECoG implantation

DBS lead placement followed a two-stage surgical procedure. During the first surgery, octopolar, directional DBS leads were stereotactically implanted into the STN after co-registration of preoperative MRI and CT. A 6-contact subdural ECoG strip (one row of 4-mm-diameter platinum contacts separated by 10 mm) was inserted through the minimally enlarged (~ 2–3 mm) frontal burr hole and advanced posteriorly toward the primary motor cortex. Across patients, the ECoG strip was positioned ipsilateral to the future implantable pulse generator (right = 13, left = 2) (Supplementary Table 2).

Both DBS and ECoG electrodes were externalized via dedicated externalization cables, allowing postoperative stimulation and recording for several days prior to internalization while patients remained on the neurosurgical ward. In a second surgery (typically 4–7 days later), DBS externalization cables were replaced with permanent cables which were tunnelled subcutaneously and connected to the subclavicular implantable pulse generator. The ECoG strip was removed through the burr hole during the second surgery.

### Anatomical localization of electrodes

Anatomical reconstruction was performed using Lead-DBS (v3.1)^41^. Preoperative MRI and postoperative CT scans were co-registered using Statistical Parametric Mapping software (SPM12)^42^, corrected for brain shift, and normalized to MNI space (Montreal Neurological Institute; MNI 2009b NLIN ASYM atlas). DBS contacts were automatically reconstructed using the PaCER^43^ algorithm and manually refined. Electrode rotational orientation was automatically corrected using DiODe v2^44^ or manually corrected based on radiopaque orientation markers.

ECoG contacts were identified manually using post-operative imaging, and their respective MNI coordinates were extracted. To ensure systematic anatomical classification, contacts were assigned to sensorimotor (primary motor and somatosensory), premotor, or parietal regions based on their proximity to the AAL3 (Automated Anatomical Labelling) parcellation atlas^45^. This atlas-based approach allowed us to categorize the electrode coverage relative to established functional boundaries, even when contact locations extended beyond the primary motor representation. Reconstructed ECoG and DBS electrode locations are shown in Fig. 1e. For spatial analyses, subthalamic evoked potentials were recorded from each STN contact in a monopolar configuration following cortical stimulation. The STN “sweet spot” was defined using previously published MNI coordinates of the dorsolateral motor STN, based on probabilistic mapping of optimal DBS stimulation sites^26^. Euclidean distances between each individual STN recording contact and the hemisphere-specific sweet spot were calculated in MNI space.

### Electrophysiological recordings

ECoG and STN-LFPs were simultaneously recorded using a TMSi Saga64+ amplifier (Twente Medical Systems International) at a sampling rate of 4000 Hz. ECoG signals were acquired from six subdural contacts, and STN-LFPs were recorded from the ipsilateral DBS lead (eight-contact directional models in all patients). All channels were hardware-referenced to the lowermost contralateral DBS contact. A large surface electrode on the right shoulder served as the ground. Bilateral surface EMG of upper limb muscles as well as ECG were recorded. Accelerometers placed at the index fingers of both hands were used to track voluntary movement. All data were stored for offline analysis and converted to the iEEG-BIDS format^46^.

### Stimulation paradigm

Recordings were performed during the externalization period while patients were comfortably seated and instructed to remain still, keep their eyes open and refrain from speaking. Patients completed recordings in two medication states: (i) ON dopaminergic therapy, taken as the patient’s usual clinical levodopa dose; and (ii) OFF dopaminergic therapy following withdrawal of all dopaminergic medication for at least 12 hours (MDS-UPDRS III OFF: 25.9 ± 9.7; ON: 16.6 ± 8.9; *p* < 0.001).

Cortical and subcortical stimulation was performed via the ECoG electrode strip and STN-DBS leads during the postoperative recording period using the Neuro Omega (Alpha Omega Engineering). ECoG stimulation contacts were selected based on the postoperative CT scan. For cortical stimulation, a bipolar configuration was used, consisting of the ECoG contact positioned over M1 and its anterior neighbour contact. This configuration maximized focal activation of primary motor cortex while minimizing current spread. Stimulation was delivered at 5 Hz, except in the first 4 patients where 1 Hz was used. An interstimulus interval that included a random pause of 1–3 s was used to prevent LTP-like effects known from rTMS experiments^47^. Each stimulus consisted of a 90 µs biphasic initial negative pulse. Prior to the main stimulation block, stimulation amplitude was slowly ramped up manually until either 10 mA or the individual motor threshold was reached, whichever occurred first. In the first four patients, stimulation was limited to a maximum amplitude of 5 mA to ensure tolerability. Importantly, this ramping procedure served only to establish the target amplitude and ensure patient comfort. These transient phases were excluded from all subsequent analyses. After this ramping procedure, stimulation continued at either 10 mA (or 5 mA in the first 4 subjects) or the motor threshold amplitude for the remainder of the recording and was delivered using a custom Python script and a Python interface for the Neuro Omega amplifier. The same maximal stimulation amplitude was used across medication states within each patient to ensure comparability. Subthalamic stimulation was performed in a bipolar configuration between the lowermost and uppermost contact of the ipsilateral DBS lead. STN stimulation was delivered at 5 mA using the same 5 Hz protocol with identical stimulation parameters. In both cortical and subthalamic stimulation sessions, efforts were taken to avoid stimulation-induced discomfort or excessive muscle activation. None of the patients reported any adverse events.

### Preprocessing

Data preprocessing and analysis was performed using custom Python (3.11.7) scripts (MNE-Python v1.8.0, NumPy v1.26.1, SciPy v1.11.3, pandas v2.0.3). ECoG and STN-LFP signals were notch filtered at 50 Hz and harmonics, high-pass filtered at 4 Hz and visually inspected for artefacts. Epochs were created from −100 to +150 ms relative to stimulus onset, using the −50 to −10 ms window for baseline correction. Epochs containing high-amplitude noise, movement artefacts, or cable-related transients were excluded. EMG traces were similarly notch filtered, band-pass filtered (20–450 Hz), baseline corrected and averaged across trials.

To distinguish physiological responses from residual stimulation artefact, a polarity reversal of the bipolar stimulation configuration was performed in one subject. While the stimulation artefact inverted its polarity and remained confined to the initial ~1 ms, the early evoked response remained unchanged. This dissociation supports the interpretation that the early response reflects a physiological signal rather than electrical artefact or passive volume conduction. The duration of the stimulation artefact was verified based on each subject’s raw signal and was consistently confined to the first ~1 ms, following stimulus onset, in line with the observations from the polarity reversal and previous literature^25^. This time window was excluded from further analysis. The onset of the earliest analysable evoked response was therefore defined as the first deflection occurring after the end of the stimulation artefact and return of the signal to baseline. The same criteria for artefact handling and definition of the earliest analysable response were applied consistently across subjects, stimulation directions, and medication states.

### Evoked response analysis

Stimulus pulse start times were detected automatically from the ECoG or DBS channels. ECoG, STN-LFP and EMG potentials were aligned according to the time point of stimulation onset. Evoked potentials were computed time-locked to cortical or subcortical stimulation events using standard averaging procedures in MNE to obtain subject-level evoked responses. Approximately 200 trials were used for each recording. ECoG and STN evoked responses were extracted using predefined time windows. The post-stimulus signal was divided into a short-latency window (1–10 ms) and a long-latency window (40–100 ms), based on prior literature^18,25^. Within each window, peak amplitude was defined as the time point of maximal deviation from baseline (either positive or negative) for each channel, subject, and condition. Peak identification was performed on single-subject, single-channel data (for spatial and clinical correlation analyses and bar plots in Figs. 2 and 3), as well as on subject-wise averages across all channels for group-level comparisons (e.g. box plots in Fig. 4). All data were evaluated blinded to channel location and medication condition, and peak identification was performed by a single rater using identical criteria across all datasets. Peak selection was restricted to time points following the end of the stimulation artefact and return to baseline, as defined in the preprocessing step. To ensure robustness of the findings and minimize potential bias introduced by peak selection, complementary statistical analyses using time-resolved cluster-based permutation testing were performed at the group level. EMG peak to peak amplitude was defined as difference between the maximum and minimum EMG voltage and latency values were characterized as the first time point, the EMG visually exceeded the baseline noise.

### Statistical analysis

Analyses were performed either at the channel level or at the patient level, depending on the research question. For spatial analyses, clinical-contact comparisons, and descriptive visualizations (e.g., bar plots of amplitude and latency (Figs. 2 and 3) and visualization of spatial distribution of subthalamic and cortical responses (Figs. 2 and 4)), single-channel values were used to capture spatial variability within patients. For statistical comparisons across patients, channel-level data were aggregated by averaging across all channels for each patient and condition, resulting in a single value per patient per condition. This approach was used for all paired and unpaired statistical tests to avoid inflation of statistical power due to non-independent channel-level observations. Differences in amplitudes and latencies were compared using paired permutation testing for the extracted amplitude and latency values and cluster-based permutation testing (1000 iterations) on the time-domain evoked potentials to control for multiple comparisons across time points. Pearson coefficient was used to test for linear correlation between EP amplitudes, latencies and patient’s MDS-UPDRS III scores. Significance threshold was set at *p* < 0.05.

### Clinical correlation

For the comparison with clinically selected stimulation settings, the clinically chosen contact was defined as the stimulation configuration used for chronic DBS therapy at the 3-month (*n* = 8) or 12-month follow-up (*n* = 7) visit. Clinical programming was performed by experienced neurologists using a standard monopolar review, in which each contact was systematically tested with respect to symptom improvement and the occurrence of side effects. While all contacts were evaluated in this structured manner, not all possible contact combinations were exhaustively tested. Clinical programming was informed by postoperative imaging and electrode reconstruction, such that the anatomical location of contacts was known to the clinician. However, final contact selection was primarily based on clinical efficacy and tolerability. Agreement was evaluated at the contact level rather than based on individual segments, as clinical stimulation was frequently delivered using co-activation of multiple segments within a level. A “level match” was defined as the contact with the largest evoked response being located on the same electrode level as the clinically selected stimulation contact(s). When chronic stimulation involved multiple active segments within a level, a match was assigned if the contact with the largest evoked response fell within the clinically selected level. As all four electrode levels were in principle available for clinical stimulation, the null model assumed equal probability of selecting any of the four levels (*p* = 0.25). Statistical significance was assessed using a two-sided binomial test.

## Supplementary information

Supplementary information

## Data Availability

Data can be made available conditionally to data sharing agreements in accordance with data privacy statements signed by the patients within the legal framework of the General Data Protection Regulation of the European Union. Requests should be directed to the lead contact, Wolf-Julian Neumann (julian.neumann@charite.de), or the Open Data officer (opendata-neuromodulation@charite.de). The data may only be used for non-commercial purposes and only to those institutions to whom the patients consented to share it. EU-based institutions need an approved cooperation agreement and joint data processing agreement with the Charité – Universitätsmedizin Berlin. The local Data Privacy Officer needs to be consulted for conducting a Data Protection Impact Assessment as provided by Charité DPO service.
