# Supplementary material for: Dopamine amplifies subthalamic long-latency responses to cortical stimulation in patients with Parkinson’s disease
Source: NPJ Parkinsons Dis. 2026 Oct 19;12:254. doi: 10.1038/s41531-026-01567-6 (PMC13680478; doi:10.1038/s41531-026-01567-6)
Supplement: Supplementary file 1 [file 41531_2026_1567_MOESM1_ESM.docx]

**Supplementary Information**

**Supplementary Table 1: Subject information.**

| **ID** | **Sex** | **DD (y)** | **LEDD (mg)** | **Preoperative UPDRS-III**  **(OFF, ON**  **levodopa; A.U.)** |
| --- | --- | --- | --- | --- |
| EL025 | M | 14 | 1990 | 72, 28 |
| EL026 | M | 12 | 1225 | 39, 4 |
| EL027 | M | 5 | 533 | 32, 20 |
| EL028 | M | 10 | 1242 | 32, 19 |
| EL029 | M | 6 | 1498 | 37, 29 |
| EL030 | M | 19 | 1250 | 56, 12 |
| EL032 | M | 6 | 1550 | 71, 8 |
| EL033 | M | 10 | 1138 | 27, 7 |
| EL034 | M | 7 | 1674 | 54, 25 |
| EL035 | M | 12 | 1550 | 21, 4 |
| EL036 | M | 7 | 1475 | 51, 8 |
| EL037 | M | 9 | 920 | 39, 20 |
| EL038 | F | 11 | 700 | 46, 19 |
| EL039 | F | 11 | 1400 | 28, 10 |
| EL040 | M | 10 | 675 | 49, 23 |

Abbreviations: DD – disease duration; F – female; LEDD – levodopa-equivalent daily dose; M – male; UPDRS – Universal Parkinson’s Disease Rating Scale

| **ID** | **DBS lead model** | **ECoG model** | **ECoG hemis-phere** | **Med. OFF** | **Med. ON** | **Recording order** | **Motor response** | **Stimu-lation frequen-**  **cy (Hz)** | **Stimu-lation amplitude**  **(mA)** | **Time since last L-dopa dose in med.ON (min)** | **Time after surgery**  **(d)** |
| --- | --- | --- | --- | --- | --- | --- | --- | --- | --- | --- | --- |
| EL025 | MT SenSight | AT TS06R-AP10X-0W6 | R | x |  | _ | no | 1 | 5 | 60 | 5 |
| EL026 | MT SenSight | AT TS06R-AP10X-0W6 | R | x * | x | ON, OFF | yes | 1 | 5 | 70 | 2 |
| EL027 | MT SenSight | AT TS06R-AP10X-0W6 | L | x | x | ON, OFF | no | 1 | 5 | 90 | 2 |
| EL028 | MT SenSight | AT TS06R-AP10X-0W6 | R | x | x | ON, OFF | no | 1 | 5 | 60 | 2 |
| EL029 | BS Vercise Cartesia | AT TS06R-AP10X-0W6 | L | x | x | ON, OFF | no | 5 | 10 | 60 | 2 |
| EL030 | MT SenSight | AT TS06R-AP10X-0W6 | R | x | x | OFF, ON | yes | 5 | 10 | 60 | 2 |
| EL032 | MT SenSight | AT TS06R-AP10X-0W6 | R |  | x | _ | no | 5 | 10 | 90 | 2 |
| EL033 | MT SenSight | AT TS06R-AP10X-0W6 | R | x | x | OFF, ON | no | 5 | 10 | 100 | 2 |
| EL034 | MT SenSight | AT TS06R-AP10X-0W6 | R | x | x | ON, OFF | yes | 5 | 10 | 60 | 2 |
| EL035 | MT SenSight | AT TS06R-AP10X-0W6 | R | x |  | _ | no | 5 | 10 | _ | 2 |
| EL036 | MT SenSight | AT TS06R-AP10X-0W6 | R | x |  | _ | yes | 5 | 6 | _ | 2 |
| EL037 | MT SenSight | AT TS06R-AP10X-0W6 | R | x | x | ON, OFF | no | 5 | 10 | 100 | 2 |
| EL038 | MT SenSight | AT TS06R-AP10X-0W6 | R | x | x | OFF, ON | yes | 5 | 7 | 60 | 2 |
| EL039 | MT SenSight | AT TS06R-AP10X-0W6 | R |  | x | _ | no | 5 | 10 | 60 | 2 |
| EL040 | MT SenSight | AT TS06R-AP10X-0W6 | R | x | x | ON, OFF | no | 5 | 10 | 120 | 2 |

**Supplementary Table 2: Recording information.**

Abbreviations: AT – Ad-Tech; BS – Boston Scientific; d – days, DBS – deep brain stimulation; ECoG – electrocorticography, MT – Medtronic, * participated only in ECoG stim recording

**Supplementary Figure 1**

***Supplementary Figure 1 Dopaminergic modulation of short- and long-latency subthalamic responses following 5 Hz cortical stimulation.*** ***a)*** *Across subject and within subject comparison of short-latency STN response reveals no significant dopaminergic modulation.* ***b-c)*** *Long-latency subthalamic response is significantly modulated by dopamine in the across subject (p < 0.001) and within subject (p = 0.017) analysis.* ***c)*** *Temporally resolved cluster-based permutation testing confirms that dopaminergic modulation is restricted to the long-latency time window. Abbreviations: ECoG – electrocorticography, STN – subthalamic nucleus, OFF and ON refers to OFF dopaminergic medication/ ON dopaminergic medication. Shaded grey area indicates cluster of significant amplitude difference between ON/OFF conditions; * p < 0.05.*

**Supplementary Figure 2**

***Supplementary Figure 2 Polarity reversal of stimulation artifact. a)*** *Schematic representation of the bipolar stimulation configuration. Bipolar stimulation was delivered between the motor cortex contact and the adjacent anterior contact.* ***b)*** *Reversal of the stimulation artifact polarity during bipolar stimulation, while the polarity of the evoked response remains unchanged. The stimulation artifact is restricted to the 0–1 ms interval, which was excluded from further analyses.*
